# Genetic, clinical and radiographic signs in knee osteoarthritis susceptibility

**DOI:** 10.1186/ar4535

**Published:** 2014-04-09

**Authors:** Luigi Minafra, Valentina Bravatà, Michele Saporito, Francesco P Cammarata, Giusi I Forte, Salvatore Caldarella, Michele D’Arienzo, Maria C Gilardi, Cristina Messa, Filippo Boniforti

**Affiliations:** 1 IBFM CNR-LATO, Contrada Pietrapollastra Pisciotto, 90015 Cefalù, PA, Italy; 2 Clinica Ortopedica e Traumatologica, Policlinico Universitario P. Giaccone-Università degli Studi di Palermo, via del Vespro 143, 90127 Palermo, Italy; 3 Nuclear Medicine, San Raffaele Scientific Institute, via Olgettina 60, 20132 Milan, Italy; 4 Department of Health Sciences, Tecnomed Foundation, University of Milano-Bicocca, via Pergolesi 33, 20900 Monza (MB), Italy; 5 Nuclear Medicine Center, San Gerardo Hospital, via Pergolesi 33, 20900 Monza (MB), Italy; 6 Unità Operativa di Ortopedia, San Raffaele Hospital ‘G. Giglio’, Contrada Pietrapollastra Pisciotto, 90015 Cefalù (PA), Italy

## Abstract

**Introduction:**

Osteoarthritis (OA) is considered to be a multifactorial and polygenic disease and diagnosis is mainly clinical and radiological. Correlation between radiographic data and clinical status has been reported. However, very few studies, especially in Caucasian people, describe the association between the Kellgren and Lawrence OA grading scale (KL) and genetic alterations to better understand OA etiopathogenesis and susceptibility. In order to update the knee OA grading, in this study we assessed the associations between KL grade, clinical features such as American Knee Society Score (AKSS), age, and polymorphisms in the principal osteoarthritis susceptibility (OS) genes in Sicilian individuals.

**Methods:**

In 66 Sicilian individuals affected by primary knee OA, the clinical and radiographic evaluation was performed using 2 sub-scores of AKSS (knee score (KS) and function score (FS)) and KL. The patients were also classified according to age. Online Mendelian Inheritance in Man (OMIM) and Database of Single Nucleotide Polymorphisms (dbSNP) Short Genetic Variations databases were used to select gene regions containing the following polymorphisms to analyze: FRZB rs288326 and rs7775, MATN3 rs77245812, ASPN D14 repeats, PTHR2 rs76758470, GDF5 rs143383 and DVWA rs11718863. Patient genotypes were obtained using Sanger DNA sequencing analysis.

**Results:**

In our cohort of patients a statistical association between the variables analyzed was reported in all associations tested (KL versus KS, FS and age). We observed that a mild to severe OA radiographic grade is related to severe clinical conditions and loss of articular function and that the severity of symptoms increases with age. Concerning the genotyping analysis, our results revealed a significant statistical association between KL grading and GDF5 rs143383 and DVWA rs11718863 genetic alterations. The latter was also associated with a more severe radiographic grade, displaying its predictive role as OA marker progression. Statistically significant association between clinical, radiographic and genetic signs observed, suggests extending the actual grading of knee OA based mainly on X-ray features.

**Conclusions:**

This work represents a multidisciplinary and translational medicine approach to study OA where clinical, radiological, and OS5 and OS6 SNPs evaluation could contribute to better define grading and progression of OA and to the development of new therapies.

## Introduction

Osteoarthritis (OA) is a multifactorial, inflammatory and degenerative disorder of the joints [1-5]. OA involves the synovial tissues and articular cartilage, resulting in symptoms that cause a decrease in the quality of life and disability. Among the risk factors, age contributes to a substantially increased risk of knee OA onset and progression [6-9], even if the association of age with the progression of knee OA is sometimes conflicting [10].

OA diagnosis is mainly clinical and radiological, and X-ray images are the gold standard to confirm the clinical diagnosis and to grade the disease [11-13]. A critical point for OA diagnosis is to identify an early onset and an early progression of this disease. Many studies analyzed the correlation between knee OA radiographic data and clinical status of the affected joint by using specific clinical scores and radiographic grading scales. A strong statistical association between pain and the Kellgren and Lawrence osteoarthritis grading scale (KL) of knee OA was observed by Muraki and colleagues in an older Japanese population [14]. Moreover, a study conducted by Cho and colleagues in a Korean population showed that knee OA symptoms became more severe with high KL levels and also that KL values were related to sex: women had worse symptoms than men at the same radiographic grade [15]. In addition, Cubukcu and colleagues investigated the possible relation between KL and the Western Ontario and McMaster Universities subscore of function, pain and stiffness, but the association was not often verified [16]. These findings were in agreement with data reported by recent observations that knee radiographic OA results cannot always be related to knee pain, due to radiographic view extension, definition of pain and variability of the cohort selected for the studies [17]. The Ahlbäck OA radiographic grading scale was used by Hernàndez-Vaquero and Fernández-Carreira in a Spanish study to analyze the relation of OA with clinical status expressed by the Hospital for Special Surgery Knee Score. However, a small variation of the clinical status score compared with the radiographic grades was observed [18].

Nowadays, molecular genetic investigations have gained an increasingly significant role in the knowledge of OA etiology and have provided evidence for a genetic component to OA [19-21]. The completion of large genome-wide association studies introduced single nucleotide polymorphisms (SNPs) as risk factors for human disease. Several gene association analyses, either genome wide or a gene candidate approach, identified different genes related to the OA susceptibility, onset and progression. OA may thus be placed into the category of polygenic diseases [22-24], but the functional influence of specific SNPs on OA needs further research in order to contribute to the etiopathogenesis. Several association studies between SNPs and OA disease remain unconfirmed or controversial, due to bias in patient enrolling criteria, differences in OA-affected joint sites, in classification and staging modes, in the radiographic evaluation scales used and in subjective differences in patient’s pain evaluation scoring. Furthermore, it becomes of interest to explore the geographical and ethnic allele distribution, which is extremely important in fully understanding the SNP variant effects. Precisely, Sicilian individuals have a specific genetic background and different allele distribution compared with the rest of Europe and with the rest of Italy (north–south genetic trend), due to distinct gene–environment interactions and, certainly, due to deep human migration movements, which have occurred in Sicily over the centuries as described by several authors [25-28].

For all these reasons and the lack of comparison with studies conducted on Italian cohorts, we have chosen to initiate this study on our cohort of Sicilian OA patients starting from loci listed and described by the Online Mendelian Inheritance in Man database, widely recognized as a comprehensive and authoritative compendium of human genes and genetic phenotypes.

Particularly, in the Online Mendelian Inheritance in Man database – which collects known genetic lesions responsible for human inherited diseases – the following principal loci of osteoarthritis susceptibility (OS) and the associated polymorphisms, SNPs and aspartic acid (D) repeats, are reported: frizzled-related protein (FRZB) rs288326 (OS1A) and rs7775 (OS1B), MATN3 rs77245812 (OS2), ASPN D14 repeats (OS3), parathyroid hormone 2 (PTHR2) rs76758470 (OS4), growth and differentiation factor 5 (GDF5) rs143383 (OS5) and DVWA rs11718863 (OS6).

In particular, the FRZB gene is a member of a family of the soluble Wingless (Wnt) antagonist. Recent evidence has demonstrated that products of Wnt and Frizzled play a key role in the development and maintenance of bones and joints [29,30]. The rs7775 and rs288326 FRZB SNPs variants showed an increased frequency in subjects with generalized radiographic OA, as confirmed by other studies in Caucasian individuals [31,32].

Articular cartilage is composed of specialized cells, the chondrocytes, that produce a large amount of extracellular matrix composed of collagen fibers. MATN3 encodes a noncollagenous extracellular matrix protein expressed during the development of the skeletal system and in the cartilage [33]. Another extracellular matrix component deregulated in the articular cartilage of OA patients is asporin protein, encoded by the ASPN gene and expressed at high levels in knee and hip cartilage of individuals with ASPN D14 repeats [34]. The PTHR2 receptor is a member of the G-protein coupled receptor family 2 and its functional role in OA is based on the observation that PTHR2 is expressed in a number of endocrine cell types and regulates pituitary hormone secretion and specifically growth hormone [30]. The role of GDF5 in the development and maintenance of bone and cartilage has also been recognized. Mutations in GDF5 are involved in several disorders of skeletal development and also in hip and knee OA progression [35-37]. Finally, the DVWA gene, which encodes for a protein containing two von Willebrand A domains, was found to harbor the rs11718863 SNP, showing a consistent association with knee OA in Japanese and Chinese OA cohorts [38].

Although several studies described the association between these specific polymorphisms and susceptibility to OA, to our knowledge no studies have examined their simultaneous presence in OA patients, especially in the European people groups. The purpose of this pilot study is to highlight possible associations between KL grade, clinical features (American Knee Society Score (AKSS), age) and the abovementioned genetic polymorphisms in order to update the knee OA grading and to improve a personalized treatment program in the future.

## Methods

### Patients

On admission to hospital, 66 Sicilian patients affected by primary OA, aged 54 to 86 years and candidates for knee surgery of arthroscopy or arthroplasty, were enrolled in this project. The patients were grouped, according to age, into two groups: young (from 54 to 65 years old) and old (from 66 to 86 years old). Among these, 61 patients were selected for genotyping analysis due to availability of a blood sample. This study (named OA_BIOMOL_1) was approved by the Ethical Committee of the San Raffaele G. Giglio Hospital, Cefalù, Italy (number of protocol: CE 2011/63) and the patients gave their written informed consent according to the Helsinki Declaration.

### Clinical evaluation

The clinical evaluation was performed for each patient before surgery using the AKSS, which includes two subscores: knee score (KS) and function score (FS). Each subscore ranges from 0 to 100 points. For KS evaluation, pain, range of motion, anterior–posterior and medio-lateral stability, flexion contracture, leg extension and varus–valgus alignment were investigated. FS evaluates knee function from a patient’s point of view, describing walking ability, climbing stairs ability and the use of walking aids. The AKSS was classified into three levels for both KS and FS: high (H_KS_, H_FS_), medium (M_KS_, M_FS_) and low (L_KS_, L_FS_).

The patients with L_KS_ and L_FS_ had scores between 0 and 49 points. The patients with M_KS_ and M_FS_ had scores between 50 and 69 points. The patients with H_KS_ and H_FS_ had scores from 70 to 100.

### Radiographic evaluation

The radiographic evaluation was performed on antero-posterior and lateral X-ray views of the knee by a single investigator using the KL, which includes four grades: grade 1, possible narrowing of joint space and possible presence of osteophytes; grade 2, definite narrowing of joint space and definite osteophytes; grade 3, definite narrowing of joint space, multiple osteophytes, sclerosis, cysts and possible deformity of bone contour; and grade 4, marked narrowing of joint space, large osteophytes, severe sclerosis, cysts and definite deformity of bone contour [39,40]. The evaluation was undertaken on an X-ray performed no more than 4 months before surgery. In our study we grouped grade 1 and grade 2 into a single grade because the radiographic differences in our cohort were considered not relevant compared with those between KL grade 3 and grade 4. The KL classification was therefore summarized into three groups: group A (grades 1 and 2), group B (grade 3), and group C (grade 4).

### Genetic analysis

The patients were genotyped by sequencing analysis, for the following genetic polymorphisms associated with OS, SNPs and D repeats: FRZB rs288326 (OS1A) and rs7775 (OS1B), MATN3 rs77245812 (OS2), PTHR2 rs76758470 (OS3), ASPN D14 repeats (OS4), GDF5 rs143383 (OS5) and DVWA rs11718863 (OS6). The Human Gene Mutation Database [41] and the dbSNP Short Genetic Variations database [42] were used to analyze gene regions containing the selected SNPs. Genomic DNA was extracted from peripheral blood using the QIAamp DNA blood mini kit, according to the manufacturer’s specifications (Qiagen Inc., Valencia, CA, USA).

After quality and quantity analysis, DNA was polymerase chain reaction amplified using primers designed by the Primer3 software [43] and listed in Table 1. Polymerase chain reaction reactions were performed with 50 ng genomic DNA in a total volume of 50 μl containing 1× PCR Gold Buffer, 1.5 mM di-MgCl_2_, 200 μM dNTPs, 200 nM forward and reverse primer mix and 1.25 U AmpliTaq Gold DNA Polymerase (Life Technologies Monza, MB, Italy). The thermal cycle profile employed a 5-minute denaturing step at 94°C, followed by 35 cycles at 94°C for 45 seconds, 59°C for 45 seconds and 72°C for 45 seconds, and a final extension step of 5 minutes at 72°C. The quality and quantity of polymerase chain reaction products were assessed on the Bioanalyzer instrument (Agilent Technologies, Santa Clara, CA, USA) and were purified using the QIAquick PCR purification kit, according to the manufacturer’s specifications (Qiagen Inc., Valencia, CA, USA).

**Table 1:** Primers sequence used for genotyping analysis

**Target gene polymorphism**	**Forward primer (5′ to 3′)**	**Reverse primer (5′ to 3′)**	**Template size (base pairs)**
FRZB (rs288326; OS1A)	cctcttggcagcaattggaac	gcccctctcccaagaaaaatg	800
FRZB (rs7775; OS1B)	agggcaggaccttgtctgtt	taagagtctgcccccaaacc	884
MATN3 (rs77245812; OS2)	tcacgtcacttcaggctgtg	tggggtctcaccatgttctc	886
ASPN (D14; OS3)	gcacattgctgaattgctttcca	ctttggggtttgctgtactttc	615
PTH2R (rs144641723; OS4)	tctcgaaccagtccctgct	cccatgacagttgctgtgg	602
GDF5 (rs143383; OS5)	gcagatgaattccaggtccag	ccatgaggtggaggtgaaga	818
DVWA (rs11718863; OS6A)	aggctgcctgccattattctt	cccatgctgtttcctttgaaca	924

FRZB, frizzled-related protein; MATN3, matrilin 3; aspn, asporin; D, aspartic acid repeat; PTHR2, parathyroid hormone 2; GDF5, growth and differentiation factor 5; DVWA, double von Willebrand A.

To perform DNA sequencing, purified amplicons were labeled with the BigDye Terminator v3.1 Cycle Sequencing Kit following the manufacturer’s standard protocol (Applied Biosystems). The thermal cycle profile employed a 1-minute denaturing step at 96°C, followed by 25 cycles at 96°C for 10 seconds, 54°C for 5 seconds and 60°C for 3 minutes. Labeled samples were purified with the X-terminator purification kit according to the manufacturer’s standard protocol and loaded in a 3500-Dx Genetic Analyzer (Applied Biosystems) for separation by capillary electrophoresis. Electropherograms and sequence files were analyzed using Sequencing Analysis and SeqScape software (Applied Biosystems).

### Statistical analysis

The association between the clinical data (KS, FS, age) and the radiographic data (KL) and the association between genotypes and KL groups (A, B, C) were analyzed using GraphPad InStat software version 3.05 [44]. The Mann–Whitney U test, the chi-square test and Fisher’s exact test were performed. Differences in groups were considered significant when *P* ≤ 0.05. Hardy–Weinberg equilibrium was evaluated as described previously [28].

## Results

### Clinical and radiographic evaluation

We recruited 66 cases (37 females and 29 males), of which 24 were young (54 to 65 years) and 42 were old (66–86 years), and they were divided into three groups (A, B, C) depending on the degree of radiographic knee OA.

According to the clinical scores we classified the patients as follows. Group A consisted of 24 patients (11 females and 13 males, 14 young and 10 old); KS was poor in 13 cases and fair in 11 cases, and the average FS score was 51 points. Group B consisted of 21 patients (15 females and six males, eight young and 13 old); KS was poor in 19 cases and fair in two cases, and the average FS score was 41 points. Group C consisted of 21 patients (11 females and 10 males, two young and 19 old); KS was low in all cases, and the average FS was 35 points.

Regarding the treatment, 22 patients of group A underwent arthroscopy and two patients arthroplasty, two patients of group B underwent arthroscopy and 19 patients arthroplasty, and 21 patients of group C underwent arthroplasty (Table 2).

**Table 2:** Clinical features and treatment for each radiographic group of patients

**KL group**	**Total**	**Females**	**Males**	**Young**	**Old**	**Arthroscopies**	**Arthroplasties**
A	24 (36.5%)	11 (29.7%)	13 (44.8%)	14(58.3%)	10 (23.8%)	22 (91.7%)	2 (4.8%)
B	21 (31.8%)	15 (40.5%)	6 (20.7%)	8 (33.3%)	13 (30.0%)	2 (8.3%)	19 (45.2%)
C	21 (31.8%)	11 (29.7%)	10 (34.5%)	2 (8.3%)	19 (45.2%)	0	21 (50%)

Data presented as number of patients (percentage). KL, Kellgren and Lawrence osteoarthritis grading scale.

According to the KS and FS scores, the patients in the L_KS_ group were in the majority (*n* = 46), while the 36 patients in the L_FS_ group generally had severe symptoms and high disability. There were 19 patients in the M_KS_ group and 23 patients in the M_FS_ group, and thus one-third of patients had moderate to severe symptoms and disability. One patient was in the H_KS_ group and seven patients were in the H_FS_ group, with zero to mild symptoms and disability (Table 3).

**Table 3:** Patient classification according to knee score and function score

**KS group**	***n***	**%**	**FS group**	***n***	**%**
L_KS_	46	70	L_FS_	36	55
M_KS_	19	29	M_FS_	23	35
H_KS_	1	2	H_FS_	7	11

FS, function score (L_FS_, low; M_FS_, medium; H_FS_, high); KS, knee score (L_KS_, low; M_KS_, medium; H_KS_, high); *n*, number of patients.

### Association between Kellgren and Lawrence osteoarthritis grading and knee score, function score and age

Association analyses were performed to verify the possible association between clinical data (KS, FS, age) and radiographic data (KL). A statistical association between the variables analyzed was observed (Table 4).

**Table 4:** Association between Kellgren and Lawrence osteoarthritis grading and knee score, function score and age

	**KL score**						
	**Group A**		**Group B**		**Group C**		***P *** **value**^**a**^
	***n***	**%**	***n***	**%**	***n***	**%**	
Low knee score	8	33.3	17	81	21	100	<0.0001
Medium knee score	15	62.5	4	19	0	0	
High knee score	1	4.2	0	0	0	0	
Low function score	8	33.3	13	62	15	71.4	0.022
Medium function score	10	41.7	7	33.3	6	28.6	
High function score	6	25	1	4.7	0	0	
Age 54 to 65	14	58.3	8	38.1	2	9.5	0.0011
Age 66 to 86	10	41.7	13	61.9	19	90.5	

KL, Kellgren and Lawrence osteoarthritis grading scale; *n*, number of patients. ^a^Chi-squared test.

#### Kellgren and Lawrence osteoarthritis grading *versus knee score*

In group A, we observed eight patients (33.3%) with L_KS_, 15 (62.5%) with M_KS_ and one (4.2%) with H_KS_. In group B, we observed 17 patients (81%) with L_KS_, four (19%) with M_KS_ and none with H_KS_. In group C, we observed all patients (*n* = 21) with L_KS_. The highest number of patients with L_KS_ were therefore in groups B and C and the radiographic findings are related to clinical pictures expressed by the KS score (*P* = 0.0001).

#### Kellgren and Lawrence osteoarthritis grading *versus function score*

In group A, we observed eight patients (33.3%) with L_KS_, 10 (41.7%) with M_FS_ and six (25%) with H_FS_. In group B, we observed 13 patients (62%) with L_FS_, seven (33.3%) with M_FS_ and one (4.7%) with H_FS_. In group C, we observed 15 patients (71.4%) with L_FS,_ six (28.6%) with M_FS_ and none with H_FS_. These data show that an increase of the OA radiographic severity corresponds to a decrease of the function score (*P* = 0.022).

#### Kellgren and Lawrence osteoarthritis grading *versus age*

In group A, we observed 14 young (58.3%) and 10 old (41.7%) patients. In group B, we observed eight young (38.1%) and 13 old (61.9%) patients. In group C, we observed two young (9.5%) and 19 old (90.5%) patients. So, it is more common to observe a medium to high OA radiographic grade in the population over 65 years old, and a low to medium in adults under the age of 65 years old (*P* = 0.0011).

### Mutational analysis of osteoarthritis susceptibility genes

The OA patients were genotyped for the following polymorphisms associated with OS, such as SNPs and D repeats: FRZB rs288326 and rs7775, MATN3 rs77245812, ASPN D14, PTHR2 rs76758470, GDF5 rs143383, and DVWA rs11718863. Percentages of the wild type, heterozygote and homozygote genotypes for each polymorphism were calculated. We reported genotyping data of the three radiographic groups (A, B, C) and the number of individuals for each genotype (Table 5). In each group, deviations of Hardy–Weinberg equilibrium for all polymorphisms analyzed were not observed.

**Table 5:** Genetic analysis results

**Polymorphism**	**Genotype**	**Group A (*n*** **= 20)**	**%**	**HWe *P *** **value**	**Group B (*n*** **= 21)**	**%**	**HWe *P *** **value**	**Group C (*n*** **= 21)**	**%**	**HWe *P *** **value**
FRZB (rs288326; OS1A)										
CC	WT	17	85	0.05	14	66.7	0.36	15	75	0.33
CT	H	2	10		7	33.3		4	20	
TT	MUT	1	5		0	0		1	5	
FRZB (rs7775; OS1B)										
CC	WT	18	90	0.03	16	76.2	0.54	13	65	0.34
CG	H	2	10		5	23.8		7	35	
GG	MUT	0	0		0	0		0	0	
MATN3 (rs77245812; OS2)										
CC	WT	19	95	0.91	20	95.2	0.91	18	90	0.81
CT	H	1	5		1	4.8		2	10	
TT	MUT	0	0		0	0		0	0	
ASPN (D14; OS3)										
D13	WT	5	25	0.65	3	14.3	0.12	4	20	0.65
D13/D14	H	11	55		14	66.7		11	55	
D14	MUT	4	20		4	19		5	25	
PTH2R (rs144641723; OS4)										
GG	WT	19	95	0.91	21	100	NA	20	100	NA
GT	H	1	5		0	0		0	0	
TT	MUT	0	0		0	0		0	0	
GDF5 (rs143383; OS5)										
TT	WT	3	15	0.18	12	57.1	0.04	7	35	0.44
TC	H	13	65		5	23.8		11	55	
CC	MUT	4	20		4	19		2	10	
DVWA (rs11718863; OS6)										
TT	WT	15	75	0.33	17	81	0.63	9	45	0.39
TA	H	4	20		4	19		10	50	
AA	MUT	1	5		0	0		1	5	

D, aspartic acid repeat; FRZB, frizzled-related protein; GDF5, growth and differentiation factor 5; H, heterozygote; HWe, Hardy–Weinberg equilibrium; MUT, homozygote; NA, not available; OS, osteoarthritis susceptibility; PTHR2, parathyroid hormone 2; WT, wild type.

### Kellgren and Lawrence osteoarthritis grading and genotype association analysis

To evaluate a potential association between genotypes, wild-type group or mutated (heterozygote and homozygote) group and the KL groups (A, B, C), the Mann–Whitney U test, the chi-square test and Fisher’s exact test were performed (Table 6). Analysis showed a statistically significant association between genotype and KL grade for the GDF5 rs143383 and the DVWA rs11718863 polymorphisms (*P* = 0.02 and *P* = 0.03, respectively). These results are in line with the study of Valdes and colleagues where GDF5 rs143383 and DVWA rs11718863 polymorphisms are consistently associated with the risk of knee OA in the Caucasian population [45], but to our knowledge this is the first study that reports the simultaneous presence of these two polymorphisms associated with KL in a European group. Unfortunately, concerning the other four OS SNPs, no genotype showed any significant association with KL data, as revealed by statistical analysis.

**Table 6:** Association between Kellgren and Lawrence osteoarthritis grading and genotype

**Polymorphism**	**KL group**	**WT**		**H + Mut**		***P *** **value**^**a**^
		***n***	**%**	***n***	**%**	
	A	17	85	3	15	0.39
FRZB-OS1A (rs288326)	B	14	66.7	7	33.3	
	C	15	75	5	25	
	A	18	90	2	10	0.17
FRZB-OS1B (rs7775)	B	16	76.2	5	23.8	
	C	13	65	7	35	
	A	19	95	1	5	0.75
MATN3-OS2 (rs77245812)	B	20	95.2	1	4.8	
	C	18	90	2	10	
	A	5	25	15	75	0.69
ASPN-OS3 (D14)	B	3	14.3	18	85.7	
	C	4	20	16	80	
	A	19	95	1	5	NA
PTH2R-OS4 (rs144641723)	B	21	100	0	0	
	C	20	100	0	0	
	A	3	15	17	85	0.02
GDF5-OS5 (rs143383)	B	12	57.1	9	42.9	
	C	7	35	13	65	
	A	15	75	5	25	0.03
DWVA-OS6 (rs11718863)	B	17	81	4	19	
	C	9	45	11	55	

D, aspartic acid repeat; FRZB, frizzled-related protein; GDF5, growth and differentiation factor 5; H, heterozygote; KL, Kellgren and Lawrence osteoarthritis grading scale; MUT, homozygote; *n*, number of patients; NA, not available; OS, osteoarthritis susceptibility; PTHR2, parathyroid hormone 2; WT, wild type. ^a^Chi-squared test.

Finally, it is possible to note in Table 6 that the DVWA rs11718863 polymorphism (genotype heterozygote + homozygote) is more represented in group C (55%), compared with the other two groups A (25%) and B (19%), suggesting that OS6 can be associated with a more severe OA radiographic grade.

## Discussion

OA grading is commonly based on the radiographic classification of Kellgren and Lawrence [39] and can be supported by general or joint specific clinical and functional scores (for example, Western Ontario and McMaster Universities score, AKSS, and so forth) [46].

The aim of our pilot study was to update the knee OA grading with further clinical and genetic associated data, since OA is nowadays considered a polygenic and multifactorial disease [47].

We investigated the possible association between KL grade, clinical features (AKSS, age) and susceptibility polymorphisms to OA, such as FRZB (OS1A and OS1B), MATN3 (OS2), PTHR2 (OS3), ASPN D14 (OS4), GDF5 (OS5) and DVWA (OS6), in order to better define the grading of this disorder. As far as we know, this is the first study that simultaneously evaluates the association between clinical and radiographic data and the presence of the known six OA susceptibility SNPs reported in the Online Mendelian Inheritance in Man database.

In our cohort of 66 patients, a statistical association between the variables analyzed was observed (KL data versus: KS, FS, age). In particular, statistical association between KL grade versus KS and FS showed that KL group A can be associated with a medium clinical score, while KL group B and KL group C are related with low KS and FS. This suggests that a mild to severe OA radiographic grade is linked to severe clinical conditions and loss of articular function. In addition, association analysis between KL grade and age of patients confirms that severity of symptoms increases with age: the majority of our patients with KL grade A were 54 to 65 years old, and most of the patients with grade C were 65 to 86 years old.

Concerning the mutational analysis, we genotyped the patients for the abovementioned OS SNPs and our results revealed a significant statistical association between KL grade and GDF5 rs143383 (OS5) and DVWA rs11718863 (OS6) genetic alterations. The presence of these SNPs could thus contribute to better define OA grading and progression.

As described by several authors, the OS5 rs143383 SNP is still the most robustly replicated polymorphism associated with OA and the only locus successfully validated across diverse Asian and European populations. The OS5 rs143383 polymorphism, localized in the 5′-untranslated region, causes a decrease in the transcriptional activity of GDF5. This gene, expressed in the adult human articular cartilage, encodes growth differentiation factor 5, a bone morphogenic protein involved in the development, homeostasis and repairing of the bone, cartilage and other articular tissues [48].

To our knowledge, genetic contribution to radiographic severity in knee OA was previously described only by Valdes and colleagues in the UK OA patients and no information is available on individuals from other European countries. For the first time, our work analyzes the association between KL grade, clinical features (AKSS, age) and the rs143383 GDF5 SNP in Sicilian OA patients. In order to support this variant role as a progression marker in knee OA, we meta-analyzed our results with those previously published by Valdes and colleagues [49]. Nonetheless, the UK OA cohorts are much larger than our Sicilian cohort but all the populations are comparable for age and body mass index, whereas a slight reduction of the rs143383 T-allele percentage is observable in Sicilian cases with respect to the other UK populations (data not shown). Overall, the Sicilian OA patients’ odds ratio of 1.53 (confidence interval, 1.11 to 2.11) describes a positive association between rs143383 GDF5 and KL (KL ≤ 2 vs. KL > 2), a trend in line with Valdes and colleagues [48], supporting this variant as an OA progression marker.

The OS6 rs11718863 polymorphism is localized in an exonic region of the DWVA gene and causes a missense mutation with a consequent amino acidic substitution Tyr169Asn [50]. The DWVA protein, involved in cellular adhesion and protein to protein interactions, interacts with β-tubulin of microtubules and has an important role in the regulation of chondrocyte differentiation, protecting articulate joints from OA onset. In particular, the OS6 rs11718863 SNP induces a decreasing interaction between DVWA and β-tubulin [51,52]. The DVWA rs11718863 SNP is reported to be strongly associated with the risk of knee OA (odds ratio = 1.43, *P* < 7 × 10^−11^) and able to influence β-tubulin binding in Asian populations. Nevertheless, Valdes and colleagues showed an association between this genetic alteration and knee OA in the UK Nottingham total knee replacement OA group (*P* < 0.046) [32]. Meulenbelt and colleagues also described a moderately significant association in the UK sample (*P* = 0.046), not confirmed for other European countries. However, the higher risk allele frequency in the European samples highlighted the ethnic different penetrance of OS genes and, once again, the need to evaluate the alleles’ geographic distribution [38].

In addition, in our cohort of patients this genetic alteration was more represented in the KL group C (55%) compared with the other groups, KL group A (25%) and KL group B (19%), respectively. We therefore suggest that OS6 can be associated with a more severe OA radiographic grade, displaying its predictive role as OA marker progression.

**Figure 1:**
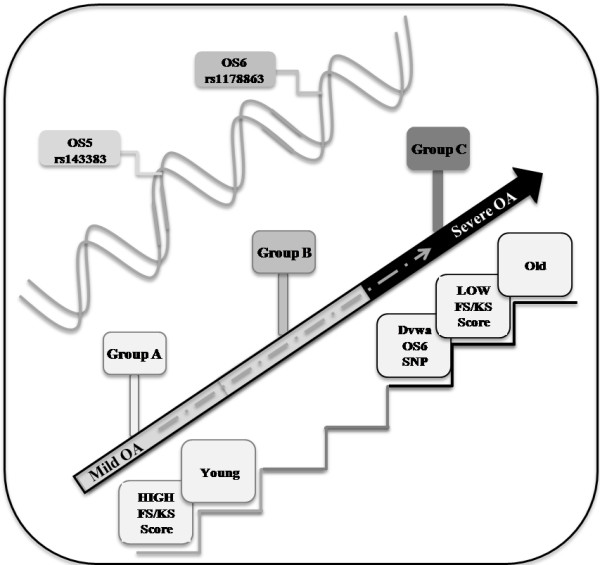
**Model of relationship between Kellgren and Lawrence osteoarthritis grading scale groups and clinical and genetic factors described in our study.** FS, function score; KS, knee score; OA, osteoarthritis; OS, osteoarthritis susceptibility; SNP, single nucleotide polymorphism.

In summary, according to our results we propose a model where we highlight the relationship between all data obtained, clinical and radiographic groups, and genetic factors. Our data pointed out that patients who belong to the KL group A (affected by mild OA) are younger and show a better joint function. In contrast, patients belonging to KL group B have a gradual progression of the disease. Finally, patients of KL group C have a more severe OA, they are older, they have poor function of the joint and most of them carry the rs11718863 OS6 polymorphism. Furthermore, according to our evidence, we show the DNA contribution, represented by rs143383 GDF5 and rs11718863 DVWA SNPs in defining the KL grade in OA prognosis and progression (Figure 1).

## Conclusion

The statistically significant association between clinical, radiographic and genetic signs observed suggests the extension of the actual grading of knee OA based mainly on X-ray features.

This work highlights the importance of a multidisciplinary and translational medicine approach to study OA where clinical, radiological, and OS5 and OS6 SNP analysis could contribute to better define grading and progression of OA and to the development of new therapies.

## Abbreviations

AKSS: American Knee Society Score; D: aspartic acid; FRZB: frizzled-related protein; FS: function score; GDF5: growth and differentiation factor 5; KL: Kellgren and Lawrence osteoarthritis grading scale; KS: knee score; OA: osteoarthritis; OS: osteoarthritis susceptibility; PTHR2: parathyroid hormone 2; SNP: single nucleotide polymorphism; Wnt: Wingless.

## Competing interests

The authors declare that they have no competing interests.

## Authors’ contributions

LM and VB were responsible for conception and design, genotyping experiments, data collection and analysis, interpretation, results elaboration, manuscript writing, and final approval of the manuscript. GIF and FPC were responsible for genotyping experiments, manuscript revision, and final approval of the manuscript. MS was responsible for data collection, patient enrolment, clinical and radiographical evaluations, manuscript writing, and final approval of the manuscript. FB was responsible for data collection, patient enrolment and clinical and radiographical evaluations, manuscript revision, financial support, and final approval of the manuscript. SC was responsible for data collection and analysis, manuscript revision, and final approval of the manuscript. MD’A was responsible for clinical evaluations, manuscript revision, and final approval of the manuscript. MCG and CM were responsible for result elaborations, financial support, manuscript revision, and final approval of the manuscript. All authors read and approved the final manuscript.
